# Developing Policy for Integrating Biomedicine and Traditional Chinese Medical Practice Using Focus Groups and the Delphi Technique

**DOI:** 10.1155/2012/149512

**Published:** 2012-05-10

**Authors:** Vincent C. H. Chung, Polly H. X. Ma, Chun Hong Lau, Sian M. Griffiths

**Affiliations:** School of Public Health and Primary Care, Chinese University of Hong Kong, China

## Abstract

In Hong Kong, statutory regulation for traditional Chinese medicine (TCM) practitioners has been implemented in the past decade. Increasing use of TCM on top of biomedicine (BM) services by the population has been followed; but corresponding policy development to integrate their practices has not yet been discussed. Using focus group methodology, we explore policy ideas for integration by collating views from frontline BM (*n* = 50) and TCM clinicians (*n* = 50). Qualitative data were analyzed under the guidance of *structuration model of collaboration*, a theoretical model for understanding interprofessional collaboration. From focus group findings we generated 28 possible approaches, and subsequently their acceptability was assessed by a two round Delphi survey amongst BM and TCM policy stakeholders (*n* = 12). Consensus was reached only on 13 statements. Stakeholders agreed that clinicians from both paradigms should share common goals of providing patient-centered care, promoting the development of protocols for shared care and information exchange, as well as strengthening interprofessional connectivity and leadership for integration. On the other hand, attitudes amongst policy stakeholders were split on the possibility of fostering trust and mutual learning, as well as on enhancing innovation and governmental support. Future policy initiatives should focus on these controversial areas.

## 1. Introduction

### 1.1. Developing Policy for Traditional, Complementary, and Alternative Medicine

Depending on the culture and history of each country, the term “traditional medicine (TM)” covers a wide variety of therapies and practices. In some cultures, the term “complementary and alternative medicine (CAM)” is commonly used to denote a similar meaning to TM. The generic term “traditional, complementary, and alternative medicine” (TCAM) is adopted by the World Health Organization (WHO) to avoid geographical variation [1]. The origin of TCAM policy development can be traced 30 years back to the Alma Ata Declaration. Promulgated in the World Health Organization (WHO) International Conference on Primary Health Care, the declaration highlighted that “*people have the right and duty to participate individually and collectively in the planning and implementation of their care, which includes access to traditional medicine*” (Section VII, Point 7) [2]. In the past decade, prevalence of TCAM use in developed countries has been on the rise [3]. Recognizing the significant role of TCAM in primary health care worldwide, the WHO has reaffirmed its support for TCAM development in the new millennium. The WHO Traditional Medicine Strategy 2002–2005 has specified the goals of (i) increasing governmental support for TCAM amongst member states and (ii) promoting integration of TCAM into member states' national healthcare systems [4]. In a WHO survey published in 2005, it was reported that 45 out of 141 responding member states have issued national policies on for TCAM. Amongst the remaining 96 states, 51 were developing relevant policies [5]. Subsequently in 2008, the *Beijing Declaration *was promulgated at the WHO Congress of Traditional Medicine. The Declaration regards TCAM policies formulation as a responsibility of governments worldwide, and has reemphasized the importance of including TCAM as part of national health systems amongst member states [6].

### 1.2. Integrating Biomedicine and Traditional Chinese Medicine: The Hong Kong Case

Health policy development is tightly intertwined with politics and societal climate. TCAM policy has no exception—evaluation of policy options requires careful consideration on social context where the relationship between TCAM and dominant biomedicine (BM) evolves. Accordingly, historical and policy analysis using case study methodology [7] is a viable first step for gaining insights on how the two paradigms may be integrated. Postcolonial Hong Kong provides an example that illustrates how the complex interplay between politics and culture influenced TCAM policy development within a BM-dominated healthcare system. During the British colonial period, the government modeled the UK NHS and established a tax funded healthcare system which was based solely on BM. On the other hand, traditional Chinese medicine (TCM) was widely used by the Chinese population as it is considered as an essential part of Hong Kong's culture. Nevertheless, TCM had no place within the public healthcare system until the reunification of mainland China and Hong Kong in 1997.

 TCM policy development is a constitutional mandate of the then newly established Special Administrative Region (SAR) government, and the major policy change was the professionalization of the TCM practitioners (TCMPs) workforce in the 2000s. This included formal statutory regulation on the practice of Chinese herbal medicine (CHM), acupuncture, bone setting, and massages by the Chinese Medicine Council of Hong Kong (CMCHK), as well as the establishment of three full-time TCM undergraduate programs in publicly funded universities [8]. Currently, new entrants to the profession are required to complete tertiary TCM training to pass a professional licensing examination, and to participate in continual TCM education programs provided by various TCM professional organizations [9]. The utilization of TCM has hiked subsequent to these policy changes [10]. Recent population wide survey reported that 19% of all private outpatient services were provided by the TCM sector. TCM users are more likely to be middle aged, female, chronically ill, experiencing lower quality of life, and having a higher or lower socioeconomic background than average [11].

While this usage pattern suggests the populations' tendency to consult both types of practitioners, policy initiatives so far have been limited to regulation rather than service redesigns that response to patients' preference. Rather than adopting mainland China's integrative organizational approach [12], the Hong Kong government has positioned TCM as a parallel profession to BM and initiatives to link BM and TCM clinicians have been minimal in the past decade. Little on TCM is being taught in the undergraduate BM curriculum, and before 1997 such element was largely nonexistent [13]. Postgraduate TCM education programs of varying depth are available to practicing BM doctors (BMD), but enrolment decisions are voluntary. Despite continual official pronouncements on TCM policy initiatives and strong support from the public, formal institutional integration of TCM into the public BM system has only been confined to 16 outpatient clinics. Of note, public subsidy on TCM is lesser than that of BM and hence fees at TCM clinics is HKD$ 120 (USD$ 15.4)—much higher than the charges of HKD$ 45 (USD$ 5.8) in their BM counterparts [14]. Their linkage with other forms of care within the public BM system is also limited, and currently there is no formal referral or coordination framework linking TCM and BM within the private sector [15].

### 1.3. Primary Care Reform in Hong Kong: An Opportunity for Fostering Integration?

Enhancement of primary care is at the top of Hong Kong's healthcare reform agenda. Major directions for reform include (i) promoting the family doctor concept; (ii) emphasizing continuity of care, holistic care and preventative care; (iii) encouraging and facilitating collaboration between BM doctors and other healthcare professionals [16]. While these reform agendas may open political windows for advancing collaboration between TCM and BM clinicians, the role of TCM in primary care is ignored in the consultation document. This omission reflects a misalignment between the government's constitutional mandate in developing TCM, healthcare policy makers' lack of motivation in fostering integration, and the population's choice for dual consultation. This misalignment may lead to fragmentation of care as patients become the only coordinators of integration [17]. For Hong Kong, formal regulation of TCMP has laid a solid foundation for enhancing coordination between the two types of care [18, 19].

Interprofessional collaboration (IPC) between biomedical doctors (BMDs) and TCAM practitioners has been regarded as a preferred mode of integration, but its implementation would require strong coordination at policy and organization levels [20]. Accordingly, discussion on how policy and organizational efforts may bridge BM and TCAM has become a topical policy issue [21]. For instance, Gaboury et al. suggested that successful integration requires common governance, payment, protocol, and management structure shared between the two services [22]. Boon and Kachan commented that IPC between the two paradigms may take different forms depending on features of health system, but they share certain keys to success. These include support and commitment from senior management, adequate resources and funding, availability of patient record sharing systems, as well as jointly agreed referral guidelines [23]. These experiences are well reflected in Sundberg et al.'s study, where his team described a successful integration model based on consensus case conference and close linkage between a gate-keeping BMD and a group of TCAM practitioners [24]. From an African perspective, Kaboru et al. reported that protection of TCAM knowledge, adequate compensation of TCAM practitioners, interprofessional education, and sufficient community participation are perquisites for IPC [25]. Furthermore, it is recommended that collaboration should start in areas that draw attention from both BM and TCAM clinicians [26].

Based on organizational sociology, the *Structuration Model of Collaboration* provided a more comprehensive framework for conceptualizing these determinants. IPC is influenced by two major dimensions: relational and organizational. On the relational dimension, success in IPC depends on shared *goals, client-centered orientation on teamwork, trust, and mutual acquaintanceship between partners*. On the organizational dimension, the degree of governance success in IPC can be operationalised by four indicators: *centrality, leadership, support for innovation, and connectivity*. on the other hand, degree of *formalization*, which clarifies how expectations and responsibilities are shared in IPC, can be reflected in two indicators: *formalization tools and information exchange* [27]. Details of these 10 indicators are listed in Table 1.

### 1.4. Aim of the Present Study

With reference to the *Structuration Model of Collaboration*, this study aims to generate potential strategies for fostering IPC between BMD and TCMP in Hong Kong, using qualitative methodology and the Delphi technique. Given the strong BM dominance within Hong Kong's healthcare ecology, lessons from Hong Kong may serve as a reference to policy makers in other healthcare systems who are tailoring integration policies to suit their local circumstances.

## 2. Method

This study consisted of two parts. The first part included a series of focus group interviews with frontline BMD and TCMP, with an aim of exploring their views on potential integration strategies. In the second part, results generated from focus group discussion were used as a blueprint for a double-round Delphi study amongst BM and TCM policy stakeholders. The Delphi process aimed to facilitate the development of a consensus-based integrative health policy proposal, and it was conducted after focus group discussion amongst frontline clinicians because: (i) introduction of potentially dominant individuals (e.g., policy makers and experts) in focus groups may lead to unwanted pressure on other participants [28] and (ii) we expected that opinions generated from focus groups would broadly reflect views from the frontline, and subsequently stakeholders would be able to ground their discussion and judgment from a “bottom-up” perspective. We assumed that the sequential combination of focus group discussions and Delphi has enabled the experts to judge, assess, and validate potential BMD-TCMP IPC strategies from a practical, local, and clinically relevant perspective. Prior to data collection, ethical approval was obtained from the Survey and Behavioral Research Ethics Committee, Chinese University of Hong Kong.

### 2.1. Part One: Focus Group Study on BMD and TCMP

Focus group discussion is an appropriate method for establishing potential strategies for integration as within group interactions would allow the exploration of diverging view amongst BMD and TCMP [29]. A purposive sample of 50 BMD and 50 TCMP were invited to attend 20 focus groups held in 2009. To ensure that participants' views were grounded more firmly to their original background, clinicians who had no prior formal education or clinical experience in integrative medicine were purposefully sampled. All focus group discussions were held separately for BMD and TCMP as homogeneous grouping would produce information in greater depth [29]. The discussion guide was developed based on the* Structuration Model of Collaboration*, as well as recent healthcare reform proposals (Table 2). Each discussion consisted of 5–8 participants and lasted for approximately 120 minutes. Proceedings were audiotaped and transcribed in verbatim. Participants were asked to provide written informed consent before the discussion starts. To enhance validity and reliability of the analysis, investigators responsible for data analysis also served as facilitators in all discussions [29]. The transcripts were analyzed for recurring themes with the aid of *NVivo 7* software [30], using the* Structuration Model of Collaboration* as a framework [31]. Specifically, transcripts were read by one investigator and possible broad themes were identified and organized under the 10 indicators of the *Structuration Model*. Meanwhile, another investigator performed the same procedure independently. The two investigators then discussed and agreed upon themes categorization. The agreed results were subsequently presented to the remaining authors and were subsequently transformed into policy statements suitable for Delphi questionnaire administration [32]. 

### 2.2. Part 2: Delphi Survey amongst Policy Stakeholders from BM and TCM Sectors

#### 2.2.1. Sampling of Experts

The Delphi process aims to obtain consensual opinions among a group of experts via a series of intensive questionnaires, interspersed with controlled feedback [33]. This part of our study aims to seek consensus on our focus group derived policy proposal from a group of heterogeneous experts via a two-round email-based Delphi process. A purposive sampling strategy was used to identify a balanced number of integration policy stakeholders from BM and TCM sectors, respectively. We defined BM stakeholders as (i) clinicians with direct experience in integrative medicine service or research; (ii) those who are responsible for the overall development of primary care policy in Hong Kong. We defined TCM stakeholders as clinicians who held official positions in major TCM professional or service organizations. The distinguishing feature of these TCM organizations was that they were accredited by the CMCHK for providing continual medical education for TCMP. In addition, given the emergence of a new generation of locally trained TCMP in Hong Kong, experts were also invited from TCM alumni associations of local universities [9]. Backgrounds of the Delphi participants are listed in Table 3. We decided to limit the number of experts to 12, and concentrated our effort in ensuring a high response rate, since increment in sample size does not lead to significant improvements in reliability when the number of experts reaches around this number [28]. Furthermore, status of participants is known to affect their contribution to a Delphi process. This is particularly important in research on integration policy, as the assumed superiority of BM experts could prevent meaningful participation from the TCM sector. To mitigate this effect, we sampled a balanced number of stakeholders with backgrounds in BM or TCM, respectively, and ensured confidentiality of individual judgments by censoring experts' identity in all correspondence.

#### 2.2.2. Delphi Survey Data Collection

Participants were initially contacted via phone or email, and they were given verbal or written information on research aims and details of the Delphi survey. The first round questionnaire, including written consent form as well as policy statements proposed by the focus group participants, was sent via email. Expert respondents were asked to rate their views on each statement on a 5-point Likert scales, ranging from strongly disagree to strongly agree [34]. They were also invited to rephrase, or contribute further information on the statements if they wished to.

#### 2.2.3. Delphi Survey Data Analysis

The returned round 1 questionnaire, which included both quantitative ratings and qualitative comments from the experts, was then analyzed to generate revised statements for reappraisal in round 2. For each statement, key feedback information in the round 2 questionnaire included (i) the respondents' own rating in round 1, (ii) median agreement rating, (iii) summary of qualitative comments, as well as (iv) whether consensus was achieved at round 1. We reported median instead of mean as it is less affected by outliers [28]. There is no agreement on the optimal cut-off figure for achieving consensus [35]. Taking into account the agreement standard adopted in published Delphi studies, the level of consensus for this study was set at 80% [36]. In other words, a statement would reach positive consensus if >80% of the panel rated agree or strongly agree; negative consensus would be reached when >80% rated disagree or strongly disagree. In round 2, participants were invited to score: (i) statements that have neither reached positive or negative consensus and (ii) any new statements proposed in the first round. In the final analysis, we calculated standard deviation values of ratings for each statement, thus the amount of disagreement amongst experts could be demonstrated [34].

## 3. Results

Focus group results generated 28 statements for our round 1 Delphi survey (Table 4). We contacted 12 experts and achieved a 100% response rate at round 1. Five statements reached positive consensus, as indicated by percentage agreement ranging from 83–92% (no. 3, no. 4, no. 15, no. 22, and no. 23, see Table 4). Their perceived importance was reflected in high median values of 5. The magnitude of disagreement amongst experts was small, as all SD values were equal to, or below 1. An exception to this applies to one statement on the need of building a non-hierarchical relationship between BMD and TCMP (no. 15, SD value: 1.16). On the other hand, one statement arrived at negative consensus, in which 92% of experts disagreed with the proposed goal of using integration as a mean for profit generation (no. 3). No new statements were proposed by the experts. In round 2, we achieved a 100% follow-up rate. Eight out of 23 remaining statements reached positive consensus (no. 2, no. 5, no. 16, no. 21, no. 24, no. 25, no. 26, no. 27, see Table 4). All median and SD values were 5 and <1, respectively, except two statements related to TCM clinical evidence and decision making (no. 24 and no. 25, SD values: 1.16).

The distribution of consensus under the *Structuration Model of Collaboration* showed a very uneven pattern. On the relational dimension, respectively, 2 statements in the *goals *(no. 2 and no. 3) and* client-centered orientation on teamwork *(no. 4 and no. 5) indicators achieved consensus, but none of the indicators within the *mutual acquaintanceship between partners *and *Trust *domains did so. On the organizational dimension, 2 statements reached consensus in each of the domains of *leadership *(no. 15 and no. 16), *Formalization Tools *(no. 24 and no. 25)* and information exchange *(no. 26 and no. 27). Three statements in the *Connectivity *domain reached consensus (no. 21, no. 22, and no. 23). On the other hand, the experts did not reach consensus on eighteen statements. It is noteworthy that none of the statements from* Centrality *or* Support for Innovation *domains reached consensus. From this pattern, it is clear that although policy ideas on *mutual acquaintance, trust, centrality* and *support for innovation *were suggested by frontline BMD and TCMP, attitude amongst integration policy stakeholders appeared to be split.

## 4. Discussion

Under theoretical guidance from the *structuration model of collaboration*, we have generated a 28 statement policy proposal on integration from focus group interviews of 50 BMD and 50 TCMP. Subsequently, we examined the acceptability of these statements amongst BM and TCM policy stakeholders. Only 13 out of 28 statements reached consensus in the Delphi process. No consensus was reached the areas of *mutual acquaintance, trust, centrality,* and s*upport for innovation. *These areas deserve deeper discussion as they could be important “rate limiting steps” when fostering integration. To promote progress in integration policy, policymakers need to facilitate consultation and discussion on areas which are contentious. Possible policy options for these areas, with reference to international literature on IPC between BMD and TCAMP, will therefore be discussed.

### 4.1. Mutual Acquaintance and Trust

Existing integration experiences suggested that interprofessional education between BMD and TCAMP is critical for mutual acquaintanceship, and subsequent referral and teamwork [37–39]. Nevertheless, stakeholders from Hong Kong seemed to be uncertain about its feasibility. For BMD, lack of knowledge is one of the most cited reasons for disapproving TCAM [40–42]. Referral to TCAMP is unlikely unless BMD are willing to gain a better understanding of TCAM, and incorporate such learning into clinical decision making [43, 44]. In Hong Kong, TCM training in BM schools could be reviewed, and future curriculum should be set at a level where BMD can gain sufficient knowledge for collaborating with TCMP [45]. Similar educational approaches can be applied in BM training courses for TCMP [46].

Although the co-location of BM and TCM clinicians is one of the most effective ways for fostering mutual acquaintance and trust [47, 48], this option may be impractical in Hong Kong as both BM and TCM primary care services are mainly provided by the private sector. As part of the healthcare reform effort, the Hong Kong government is planning to launch a family doctor register that provide a list of “private doctors who serve as family doctors and provide comprehensive primary care to patients.” It is proposed that the register will include doctors' basic qualifications as well as their continual professional development participation record. This information is supposed to guide the public and help them to “identify those who are practicing as family doctors from those who would like to pursue a practice in other specialty area, and to provide patients with adequate information that will facilitate them to choose the provider” [49]. To this end, a family doctor list could be a viable way for creating an IPC network between BMD and TCMP. Clarifying each primary care providers' willingness and competency in IPC can reduce uncertainty in referral decision making for both sectors, as well as enabling patients to choose clinicians that are willing to collaborate with practitioners from other paradigms. This may in turn promote IPC within the private sector, as this could attract patients who would like to receive both BM and TCM treatment in a coordinated manner.

### 4.2. Support for Innovation and Centrality

Redistribution of responsibility across professional boundaries is a defining feature of effective integration. The practice of acupuncture by BMD, as well as the rights to order BM diagnostic test by TCMP are examples of cross fertilization between the two paradigms. While these initiatives could foster innovation in their respective fields, the TCM sector may perceive the use of acupuncture by BMD as an explicit absorption of TCM modalities into the BM tool box, and in the longer term this may sideline the role of TCMP [50]. Meanwhile, BMD may see their monopoly in ordering diagnostic tests being challenged. The potential and political feasibility of resolving this interest laden dilemma could be higher in the public sector within Hong Kong, but stakeholders are yet to reach consensus on how TCMP should be employed in the publicly funded health system. Poor employment prospects amongst TCM graduates in Hong Kong could be a major barrier to integration [9]. International experience has shown that securing funding for launching and sustaining integrative medicine services is a major challenge for health authorities before the gains of integration can be harvested [48, 51]. This challenge is particularly acute when it comes to the issue of generating sufficient funding for recruiting experienced, qualified TCAMP [48]. The mode of employment carries strong implications on TCAMP's sense of belonging within a predominantly BM institution, as well as on their intention to work collaboratively with BMD [42, 52]. When TCAMP are employed on a part time fee for service basis, they are often reluctant to perform communication and team building activities like case conferences, since many TCAMP may feel that they are not remunerated for such activities [51, 53].

### 4.3. Future Research Directions

Two further critical policy issues deserve deeper debate. Firstly, stakeholders in this study have not reached consensus on the need for a dedicated knowledge translation and dissemination center for supporting integration. Superficially, reasons for disagreement may relate to *whom and where* TCM research should be done, but the *how* question should also be considered carefully. While it is acknowledged that clinical evidence could be a potential tool for bridging the two paradigms, a hierarchical view on evidence may have limited applicability in evaluating TCAM. To balance the demand for both internal and external validities, a circular perspective on evidence could provide a rich toolbox of research methods that will contribute to the evidence base of TCAM [54]. The importance of policy makers' perception on TCAM's evidence base has been partially reflected in our stakeholders' disagreement on the equalization of users' charges for TCM and BM outpatient services. A changing perspective on evidence may have a significant impact on policy makers' decision on how should TCAM be financed in a tax funded healthcare system. The linkage between perception on evidence and financing decision would warrant further investigations.

Secondly, with the future implementation of electronic health system worldwide, leaders of integrative medicine services may consider the addition of a feedback system that provides BMD and TCAMP with timely updates on referral outcomes. BMD may fill their knowledge gaps in TCAM by receiving comments from patients and colleagues via such a system [55, 56] and repeated positive feedback may encourage BMD to refer patients with an increasingly wider range of problems [40, 56]. Meanwhile, TCAMP may make use of this mechanism to gain higher credibility within the BM dominated health system [48]. on long run, evaluation of IPC should be formalized, focusing on outcomes that are recognized by both BMD and TCAMP [55].

### 4.4. Strengths and Weaknesses of This Study

In our focus group study, we sampled the views of a broad range of BMD and TCMP who has direct involvement with care provision. However, we cannot eliminate self-selection bias in the recruitment process as those who were more willing to attend discussions are often holding stronger opinion for, or against, integration. While the resultant bias is difficult to quantify, our qualitative approach has enabled an in-depth, diversified understanding on key issues surrounding integration across the two disciplines. This provided a strong foundation to our subsequent Delphi study. In this second part, panel members were selected carefully to represent a wide heterogeneous range of expertise from different settings. While this provided a broad perspective on our final findings, it may also lower the number of consensus reached as experts may find little common ground on the contentious issues of integration policy [57]. We achieved a 100% response rate for both rounds, but our expert sample may not be representative to all integration policy stakeholders in Hong Kong. Nevertheless, a larger sample size may pose difficulties in ensuring high response rate, and excessive variations in judgment could be problem when the number of experts is larger than 20 [57].

## 5. Conclusion

While the WHO Beijing Declaration is advocating the integration of BM and TCAM, many member states may consider this as a second step subsequent to formal regulation of TCAM practice. With a decade's experience in regulating TCM, Hong Kong represents a unique case for demonstrating how integration, as defined as IPC between BMD and TCAMP, could proceed after regulation. The current consensual policy proposal can be considered as a starting point for fostering IPC between the two paradigms of practice. Findings from this study can be a useful reference for policy makers in other countries. However, given the special circumstance in Hong Kong where TCM development is considered as a constitutional mandate, we have taken an advocacy approach in conducting the present study. We may have overlooked certain barriers to integration, and thus the external validity of our findings should be carefully evaluated via future comparative studies.

## Figures and Tables

**Table 1:** Structuration model of collaboration.

Relational Dimensions	Indicators	Description
(1) Shared goals and vision	Goals	(i) Consensual and comprehensive goals shared by the professions (ii) Essential point of departure for a collaborative undertaking
	Client-centred orientation on teamwork	(i) Differences between professionals lead to asymmetry of interests amongst partners or a partial convergence of interests (ii) Private interest will emerge of shared goals are not negotiated, resulting in opportunistic behavior and a concomitant loss of focus on client-centered collaboration

(2) Internalization	Trust	(i) Collaboration is possible only when they have trust in each other's competency (ii) When there is too much uncertainty, professionals tend to avoid collaborating (iii) Results of collaboration are used to evaluate each other and build trust
	Mutual acquaintanceship	(i) Professionals must know each other's values, level of competence, disciplinary frame of reference, and approach to care and scope of practice if they are to develop a sense of belonging to a group and succeed in setting common objectives (ii) It is necessary to create the social conditions that will foster collaboration, particularly through social interaction

Organizational Dimensions	Indicators	

(3) Governance	Centrality	(i) Centrality refers to the existence of clear and explicit direction towards collaboration between professions (ii) Central directives are essential strategic and political tools that help to materialize the implementation of collaborative processes and structures
	Leadership	(i) Frontline leadership is essential for the success of IPC. Power differential between partners should be minimized (ii) Power should not be concentrated in the hands of a single partner; all partners must be able to have their opinions heard and to participate in decision making
	Support for innovation	(i) Collaboration often involves dividing responsibilities differently between professionals and between institutions. It necessarily entails innovations in clinical practices and in the sharing of responsibilities between partners (ii) Interprofessional learning and expert support is essential for implementation these innovations
	Connectivity	(i) Strong connectivity allows for rapid and continuous adjustments to problems arising from coordination (ii) It takes the form of information and feedback systems, committees, and so forth

(4) Formalization	Formalization tools	(i) They are means of clarifying the various partners' responsibilities and negotiating how responsibilities are shared (ii) For professionals, it is important to know what is expected of them and what they can expect of others
	Information exchange	(i) Refers to the existence and appropriate use of an information infrastructure that allow for rapid and complete exchanges of information between professionals (ii) Feedback provides professionals with the information they need to follow up with patients as well as to evaluate their partners on the basis of the quality of the written exchanges and feedback

**Table 2:** Focus group discussion guideline.

Topic 1	Integrating biomedicine and traditional Chinese medicine
A	How would you define integration? Can we reach a consensus on its definition? (Probe: agree? Disagree? Why? In which aspect?)
B	If this definition were to be applied Hong Kong, what sort of changes should be made? How? (Probe: link to contextual factors in local healthcare system)
C	Do you have any thoughts on adding more content into this definition? (Probe: you may refer to the notes written before. Write down the comments on a display board.)
D	Can you explain your ideas further the reasons for your answers with other members of the group?
E	How about disagreement? (Probe: facilitate debate among members. Inviting those who are silent to comment on the new ideas)

Topic 2	Evolving relationship between BMD and TCMP

A	At what stage do you think integration has reached locally?
B	How far do you think that shows satisfactory progress? (probe: why agree? Why disagree?). Can you design a blueprint for progress?
C	How far do you think the pathway towards integration outlined here is appropriate for Hong Kong? (probe: why agree? Why disagree?)
D	What changes in the pathway might you consider more appropriate to the local system? How? (probe: link to contextual factors in local healthcare system)
E	Can you explain your ideas further with other members of the group?

Topic 3	Knowledge, attitude, and skills for individual BMD and TCMP in the process of integration

A	How far do you consider our local clinicians have acquired all the knowledge, attitudes and skills necessary for integration? Why? Why not?
B	List all the knowledge, attitudes, and skills that you believe to be necessary for integration. Focus on the requirements for your profession only
C	Have you have any comments on the requirements for the profession other than yours (i.e., BMD comments on requirement for TCMP and vice versa)
D	Do you think these requirements feasible under local healthcare setting? Why? How far do you consider the list is reasonable? Any final additions or deletions?

**Table 3:** Background of the Delphi participants.

Stakeholders from the BM sector	Stakeholders from the TCM sector	
(1) A senior manager in integrative BM-TCM service working within public healthcare sector	(1) One representative from four TCM professional organizations responsible for providing accredited continual medical education to TCMP	
(2) A senior government official in charge of primary care policy		
(3) A leader in primary care in Hong Kong as well as the Asia pacific region		
(4) A BM academic with expertise in TCM research		
(5) A leader in BM's specialist standard setting body	(2) Two representatives from alumni associations of local, tertiary trained TCMP	
(6) A BM academic in primary care		

**Table 4:** Statements of Delphi survey.

Statements	Round 1			Round 2		
	Median (SD)	Agreement (%)	Consensus (Yes/No)	Median (SD)	Agreement (%)	Consensus (Yes/No)
Shared goals and vision: (i) goal						

(1) The most important goal in developing IPC between BMD and TCMP is to respect patient's choice for both types of medicine	4 (1.08)	58	No	4.5 (0.87)	75	No
(2) The most important goal in developing IPC between BMD and TCMP is to facilitate evidence based research on the efficacy and safety of TCM and integrative medicine (IM) treatments	5 (1.07)	75	No	5 (0.80)	83	Yes
(3) The most important goal in developing IPC between BMD and TCMP is to generate profit by satisfying existing patient demand.	1 (1.00)	8	Yes (negative consensus)	N/A		

Shared goals and vision: (ii) client-centered orientation on teamwork						

(4) Stronger personal contribution is expected in the healthcare financing reform Hence the public should have the right to choose between BM and TCM when utilizing health services	5 (1.00)	83	Yes	N/A		
(5) BMD should respect patients' choice for BM-TCM shared care in the in-patient environment.	5 (1.27)	75	No	5 (0.79)	83	Yes
(6) The current charges for public BM general outpatient clinics and TCM clinics are HKD$ 45 and HKD$ 120, respectively. Fees for both types of clinics should be equalized	3 (1.56)	33	No	3 (1.00)	33	No

Internalization: (i) Mutual acquaintanceship						

(7) TCM should be incorporated into the BM curriculum as a compulsory element	2.5 (1.44)	42	No	3.5 (1.24)	50	No
(8) BM training should be strengthened in current TCM curriculum	3 (1.41)	42	No	3 (1.19)	25	No
(9) Dual degree course on both BM and TCM should be made available locally at undergraduate level	4 (1.83)	58	No	4 (1.03)	75	No

Internalization: (ii) Trust						

(10) The willingness and competency of a BMD in referring to TCM should be indicated in the family doctor list	2.5 (1.38)	25	No	3 (1.06)	33	No
(11) Variation in existing TCMP's competency is a major barrier for referral by BMD. The Chinese Medicine Council of Hong Kong should designate which TCMP are competent to receiving BMD referral.	2 (1.73)	33	No	3 (1.38)	25	No

Governance: (i) centrality						

(12) The regulatory bodies, Hong Kong Medical Council, and the Chinese Medicine Council of Hong Kong should be merged to facilitate IPC	2 (1.50)	17	No	2.5 (1.66)	33	No
(13) Local TCM undergraduate courses have become unpopular in recent years due to poor graduate employability. Such training should be reviewed	3.5 (1.24)	50	No	4 (0.79)	58	No
(14) A better career prospects to Hong Kong graduates should be offered to maintain local TCM talent pool	3 (1.75)	42	No	4 (1.44)	58	No

Governance: (ii) Leadership						

(15) In the development of integrative BM-TCM services, existing TCMP and BMD should Collaboratively work as equals without an assumed hierarchy	5 (1.16)	92	Yes	N/A		
(16) Part-time formal TCM training leading to qualifications recognized by both Medical Council and Chinese Medicine Council should be offered to BMD	4.5 (1.28)	67	No	5 (0.98)	83	Yes
(17) Part-time formal BM training leading to qualifications recognized by both Medical Council and Chinese Medicine Council should be offered to TCMP	3 (1.68)	42	No	3 (1.00)	42	No

Governance: (iii) support for innovation						

(18) Currently, the three schools of Chinese Medicine focus on laboratory research. A health service research agency for evaluating effectiveness and cost effectiveness of TCM and IM should be set up	3 (1.21)	25	No	3 (0.89)	42	No
(19) With appropriate training, TCMP should be allowed to order BM diagnostic tests	4 (1.13)	75	No	4 (0.74)	75	No
(20) With training accredited by the Chinese Medicine Council, BMD should be allowed to perform acupuncture	5 (1.64)	67	No	5 (0.89)	75	No

Governance: (iv) connectivity						

(21) BMD and TCMP working within the private sector should be encouraged to practice IPC and shared care	5 (1.54)	75	No	5 (0.65)	92	Yes
(22) BMD and TCMP working within the public sector should be encouraged to practice IPC and shared care	5 (0.90)	92	Yes	N/A		
(23) Public BM sector should consider and accept, if appropriate, referral from private sector TCMP	5 (0.90)	92	Yes	N/A		

Formalization: (i) Formalization tools						

(24) Under the requirement of evidence based medicine, high quality clinical evidence on many TCM modalities is not available. TCM can be added to BM treatment as long as such addition is not found to be harmful	4.5 (1.40)	58	No	5 (1.16)	92	Yes

(25) Under the requirement of evidence based medicine, high quality clinical evidence on many TCM modalities is not available. Thus TCM must be used separately from BM treatment	4.5 (1.61)	58	No	5 (1.16)	92	Yes

Formalization: (ii) information exchange						

(26) To facilitate interpretation of TCM medical records by BMD, consulting services by a dual-trained BMD-TCMP should be offered	5 (1.51)	67	No	5 (0.67)	92	Yes
(27) To facilitate interpretation of BM medical records by TCMP, consulting services by a dual-trained BMD-TCMP should be offered	4.5 (1.21)	67	No	5 (0.67)	92	Yes
(28) The design of electronic health record system should be able to present and synthesize both BM and TCM records.	3 (1.67)	42	No	3 (1.15)	42	No

BM: biomedicine; BMD: biomedicine doctors; TCM: traditional Chinese medicine; TCMP: traditional Chinese medicine practitioners; IM: integrative medicine; N/A: not applicable as consensus has already been reached.
